# Immunotherapy for Peritoneal Carcinomatosis: Challenges and Prospective Outcomes

**DOI:** 10.3390/cancers15082383

**Published:** 2023-04-20

**Authors:** Mefotse Saha Cyrelle Ornella, Narayanasamy Badrinath, Kyeong-Ae Kim, Jung Hee Kim, Euna Cho, Tae-Ho Hwang, Jae-Joon Kim

**Affiliations:** 1 Department of Pharmacology, School of Medicine, Pusan National University, Yangsan 50612, Republic of Korea; ornella@pusan.ac.kr (M.S.C.O.);; 2 Bionoxx Inc., Parkview Tower #1905, 248 Jeongjail-ro, Bundang-gu, Seongnam 13554, Republic of Korea; 3 Division of Hematology & Oncology, Department of Internal Medicine, Pusan National University Yangsan Hospital, Yangsan 50612, Republic of Korea

**Keywords:** intraperitoneal immunotherapy, peritoneal carcinomatosis, CAR-T cells, vaccines, ascites, carcinoembryonic antigen, dendritic cells, catumaxomab

## Abstract

**Simple Summary:**

Peritoneal carcinomatosis is a challenging condition that affects many cancer patients, and conventional therapies have limited efficacy in treating it. However, recent advances in the field of immunotherapy have shown promise in improving treatment outcomes. One promising approach is immune checkpoint inhibitors, which block proteins that inhibit T-cell activity and promote an anti-tumor immune response. Another approach involves the use of CAR-T cells, which are genetically modified T cells engineered to recognize and target cancer cells expressing specific antigens. In addition, dendritic cells and vaccine-based therapeutics are also designed to stimulate the immune system to recognize and attack cancer cells. The authors also discuss the potential benefits of combining different immunotherapeutic approaches to improve treatment efficacy. While there is still much to be learned about the use of immunotherapy for peritoneal carcinomatosis, the available evidence suggests that it holds promise as a potentially effective and well-tolerated treatment option.

**Abstract:**

Peritoneal metastasis, also known as peritoneal carcinomatosis (PC), is a refractory cancer that is typically resistant to conventional therapies. The typical treatment for PC is a combination of cytoreductive surgery (CRS) and hyperthermic intraperitoneal chemotherapy (HIPEC). Recently, research in this area has seen significant advances, particularly in immunotherapy as an alternative therapy for PC, which is very encouraging. Catumaxomab is a trifunctional antibody intraperitoneal (IP) immunotherapy authorized in Europe that can be used to diminish malignant ascites by targeting EpCAM. Intraperitoneal (IP) immunotherapy breaks immunological tolerance to treat peritoneal illness. Increasing T-cell responses and vaccination against tumor-associated antigens are two methods of treatment. CAR-T cells, vaccine-based therapeutics, dendritic cells (DCs) in combination with pro-inflammatory cytokines and NKs, adoptive cell transfer, and immune checkpoint inhibitors are promising treatments for PC. Carcinoembryonic antigen-expressing tumors are suppressed by IP administration of CAR-T cells. This reaction was strengthened by anti-PD-L1 or anti-Gr1. When paired with CD137 co-stimulatory signaling, CAR-T cells for folate receptor cancers made it easier for T-cell tumors to find their way to and stay alive in the body.

## 1. Introduction

Peritoneal carcinomatosis (PC), a fatal tumor diagnosis, often has a dismal prognosis. PC is the metastatic involvement of the peritoneum, the thin membrane that encircles abdominal organs [1,2]. In gastrointestinal (e.g., colorectal and gastric cancers) and gynecological (e.g., ovarian cancer) malignancies, the average survival time for PC is less than six months [3,4,5,6,7]. PC occurs in around 15% of colorectal cancer patients [2] and up to 50% of patients with recurrent gastric cancer [8]. Patients with PC have limited therapeutic options. The specific etiology of the ailment is unknown, and the optimal target has not yet been identified. Palliative systemic treatment is the first line of therapy since a full surgical intervention is tricky and often results in an increase in morbidity and death [9]. Chemotherapy or other types of permitted systemic therapy are typically insufficient for peritoneal dissemination. This is due to restricted drug delivery and adverse effects that include intestinal obstruction and abdominal bloating. Multiple studies have demonstrated that PC patients who have cytoreductive surgery (CRS) and hyperthermic intraperitoneal chemotherapy (HIPEC), especially when full cytoreduction is achievable, have a higher survival rate [10,11,12]. However, the majority of clinical research on peritoneal surface cancers is hindered by chronically high recurrence rates and poor patient survival [13]. Most patients have extensive peritoneal metastases and severe disease, and the location of the tumor growth on the peritoneal layer inside the abdominal area is one of the characteristics that separate PC from distant metastases. Consequently, locoregional treatment alternatives are likely more suited for treating PC. In response to the difficulties presented by PC in understanding genetics and the formation of cancer, the value of cutting-edge tools such as RNA sequencing and cytometry has grown [14,15].

Immunotherapy has emerged as a promising treatment approach for PC. The peritoneal cavity contains a variety of immune cells, and recent research has shown that the immune system plays an important role in controlling tumor growth in the peritoneum. However, tumors in the peritoneum often develop mechanisms to evade the immune system, leading to disease progression and poor outcomes [16].

As a result, substantial efforts have been undertaken to develop a new immunotherapeutic approach that can enhance immune cell trafficking into the PC and tumor immunogenicity. In this review, we discuss how immunotherapy works and how recent preclinical and clinical research shows that immunotherapy is the best method of treating PC.

## 2. Peritoneal Carcinomatosis

### 2.1. Peritoneum and Peritoneal Carcinomatosis

**Figure 1:**
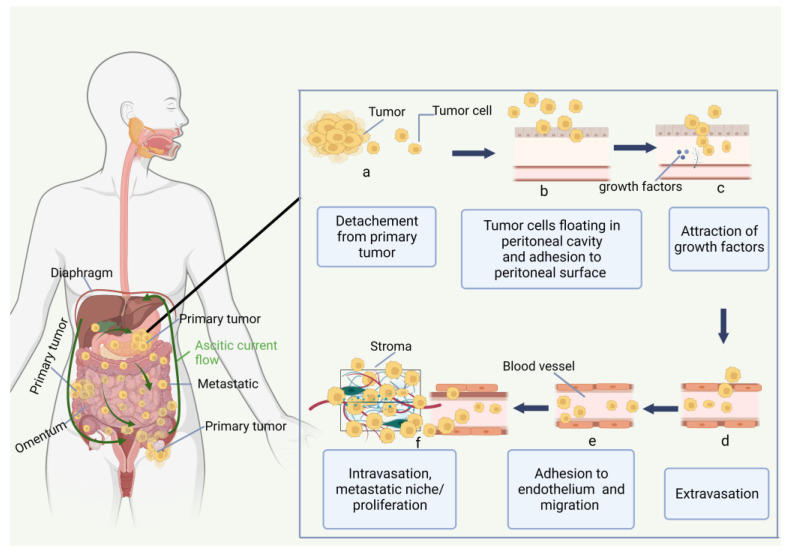
The development of peritoneal carcinomatosis: (**a**) The cancer cells dissociate from the main tumor and (**b**) penetrate the epithelial tissue. Cancer cells that have exfoliated invade the underlying basal lamina and stroma. (**c**) Stromal cancer cells defy apoptosis and entice growth substances that stimulate proliferation and angiogenesis. (**d**) After breaking through the endothelial cells that border the vessels, cancer cells spread to other parts of the body. (**e**) Blood vessels in close proximity to a tumor facilitate the development of distant metastases. (**f**) At a future metastatic site, cancer cells adhere to and invade the endothelium to form a new metastasis.

One of the most active tissues in the human body is the peritoneum, often known as the surface tissue of the peritoneal cavity. Immunological mechanisms are most likely to manifest in individuals with local or diffuse peritonitis caused by any bacterial infection. The peritoneum has several transport systems, substantial microcirculation, and substantial blood flow. It is an important barrier that prevents intra-abdominal diseases from spreading throughout the body [17]. A multistep procedure that leads to the development of PC begins with the separation of malignant cells from the early tumors. The detached cells then attach themselves to peritoneal mesothelial cells, causing these cells to shrink and expose the basement membrane. The cancerous cells then multiply and form a cluster, which triggers the process of angiogenesis. The newly formed cluster, along with the blood vessels surrounding the tumor, supports the growth of the tumor, as shown in Figure 1 [18].

In 1979, Sugarbaker explained that the molecular connection and compatibility between receptors on cancer cells and ligands on host cells are responsible for organ-specific metastasis [19]. One more illustration of peritoneal malignancies can be understood through Stephen Paget’s “seed and soil hypothesis.” A malignant tumor releases cells (seeds) that disperse randomly but can only live and proliferate in tumor-accepting localizations (soil). This helps to clarify why cancer cells from the digestive tract, ovaries, and stomach tend to congregate in the peritoneum [20].

Primary and secondary PC are two forms of this cancer that can appear on the peritoneal surface. Cancer of the peritoneum and mesothelioma of the peritoneum are the most prevalent types of primary peritoneal tumors. Secondary tumors in the peritoneum include those of the digestive tract and the gynecological system, to name just two. PC has traditionally been considered a kind of systemic and extensive metastasis, as well as the ultimate stage of illness for which only palliative treatment is warranted [21].

However, in its earliest stages, the disease may cause no symptoms at all. Ascites or intestinal obstruction are common early symptoms, which often indicate a more advanced stage of the illness with a larger tumor load that is more challenging to cure [22]. Detecting PC early, while there is still just a small amount of tumor, may improve the success rate of existing treatments [23].

The use of CRS followed by HIPEC (CRS-HIPEC) has shown some effectiveness, but only in a few patients with light disease loads. All detectable intraperitoneal (IP) tumors are removed with CRS-HIPEC, and any remaining microscopic disease is treated with chemotherapy administered locally. Compared to systemic chemotherapy, individuals with CRC PC who had CRS-HIPEC had a considerably higher 5-year survival rate [12,24].

Most people with PC are not good candidates for CRS-HIPEC, hence their condition usually worsens and poor survival [2,25]. However, the success of CRS-HIPEC in PC shows that treatments given locally may be able to meet this important unmet clinical need.

### 2.2. Immune Environment of Peritoneal Carcinomatosis

**Figure 2:**
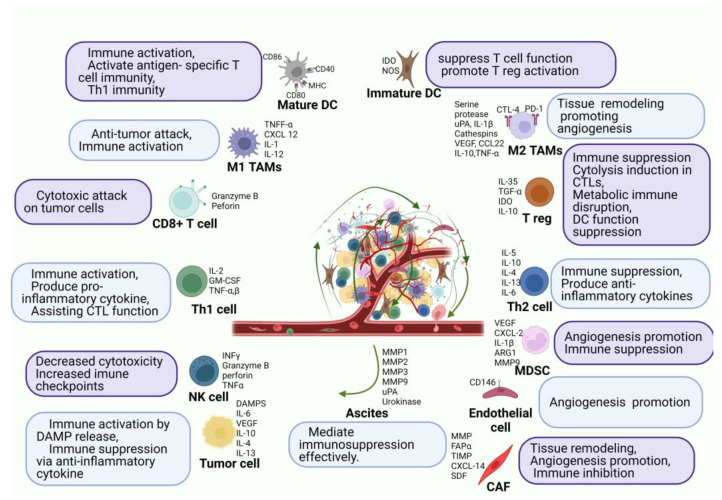
Cells in the ascites tumor microenvironment (TME). The peritoneal carcinomatosis microenvironment is formed of malignant ascites and solid tumor tissue. Under the influence of the ascites, which exchange O2 and nutrients with the circulatory system, cells actively communicate with each other through molecules they produce (cytokines, chemokines, DAMPs, etc.) and receptors they express (MHC, PD-1). The TME may re-polarize the same set of cells or move cell components to other locations. The growth or shrinkage of a tumor site is controlled by a complex network of cells and molecules in the TME.

The innate (neutrophils, macrophages, dendritic cells, and natural killer cells) and the adaptive (B and T lymphocytes) immune systems can recognize and destroy tumor cells. However, cancer cells gain the ability to evade immune surveillance by targeting or manipulating the immune system. Since lymph nodes and the greater omentum both include immune cells such as macrophages and lymphocytes, immune cell activation may be a potential PC treatment strategy [16]. The peritoneal cavity has immunologically competent cells, such as 45% of monocytes/macrophages (CD68+), 45% of T-lymphocytes (CD2+), 8% of NK-cells (natural killer cells), and 2% of dendritic cells, as well as A substantial proportion of CD4+ (92%) and CD8+ (73%). Approximately 49% of the cells in the peritoneum were positive for class II major histocompatibility complex antigens [26]. In contrast to CD45RO-naive T-lymphocytes, CD45RO+ T-lymphocytes have already differentiated into memory and effector T cells. In contrast to peripheral blood cells, which have a predominance of CD8+ T-lymphocytes, healthy individuals have an inverted CD4+/CD8+ T-lymphocyte ratio. Innate immunity is activated by mesenchymal precursors of the peritoneum. Interleukin-1 (IL-1), interleukin-6 (IL-6), prostaglandin E2, granulocyte stimulating factor (GCSF), granulocyte monocyte colony-stimulating factor (GM-CSF), monocyte colony-stimulating factor (MCSF), and vascular epithelial growth factor (VEGF) are all pro-inflammatory mediators that are released by mesenchymal cells. Figure 2 shows the cell ascites tumor microenvironment (TME).

In vitro experiments revealed that, in response to interferon-gamma stimulation, peritoneal mesothelial cells express HLA-DR and ICAM-1 molecules. These findings demonstrate that antigen presentation to T cells is more potent than previously believed, hence promoting the proliferation of T cells driven by anti-CD3. The production of interleukin-2 (IL-2), interleukin-15 (IL-15), and interferon-gamma indicate the presence of a favorable cytokine environment. In this regard, it has been proposed that stimulating the innate immune system is an effective therapy for peritoneal dissemination. This may be accomplished with dendritic cells. Because they are antigen-presenting cells, they might be used as therapeutic vaccines in co-culture with autologous T lymphocytes to educate and stimulate specific antitumor lymphocytes [27]. Moreover, macrophages can stimulate Th-1 immune responses [28] or gene therapy using the intercellular adhesion molecule of the adenovirus vector vehicle (ICAM-2), which results in NK infiltration in peritoneal metastatic (PM) lesions [29] by attaching to the Toll-like receptor on antigen-presenting cells.

The tumor immune microenvironment (TME) of PM lesions in gastric cancer is different from primary lesions. Fujimori et al. described that the PM tumor is an enriched desmoid (fibrous) component induced by CAFs and the number of CD8 positive cells was significantly lower in PM lesions than in primary lesions. Conversely, the number of CD163 positive cells (M2 macrophages) was significantly higher in PM lesions than in primary lesions. Therefore, PM mouse models should be used and established similar to the TME of human clinical PM in gastric cancer by using YTN16 and LmcMF [30]. Additionally, approaches for immune-suppressive cells, such as M2-like macrophages connected to PD-L1 expression in gastric cancer cells [31] or Tregs with intraperitoneal arsenic trioxide (AS_2_O_3_), are the subject of investigation [32]. The immunity of the peritoneal compartment is physiologically in an anti-inflammatory state. T-cell induction, antigen presentation, and rapid immunological activation are nevertheless possible upon exposure to pathogenic antigens. Immunotherapeutic intervention is particularly attractive due to the unusually high level of local immunological competence in the peritoneal compartment [17].

Immunotherapy is generally known to be less effective in PC due to the characteristics of immunologic change, such as an immunosuppressive environment created by the tumor, which hinders the activation of immune cells against cancer cells [33,34,35,36]. However, recent studies have shown promising results by using immunotherapeutic strategies to activate the immune cells infiltrating the peritoneal area. Therefore, although immunotherapy may have limited effects on PC, the activation of immune cells through various strategies could potentially improve outcomes in patients with this condition.

## 3. Immune Checkpoint Inhibitors

Patients with a wide variety of cancers benefit from antibodies that target immunological checkpoints, such as cytotoxic T-lymphocyte-associated protein-4 (CTLA-4), PD-1, and PD-L1 [37]. In 2011, for the first time, the FDA approved ipilimumab as an immune checkpoint inhibitor (ICI) for the treatment of metastatic melanoma [38]. Since then, six other ICIs have received FDA approval for use in the United States [39] and many more are now undergoing testing in humans as shown in Table 1. However, these ICIs have low success rates when used alone to treat PC [40].

ICI has shown excellent and, most importantly, long-lasting responses in advanced tumor patients, unlike targeted treatment and chemotherapy. It is known that factors such as microsatellite instability (MSI-H), tumor mutation burden (TMB), and PD-L1 expression can be used to predict the therapeutic response of an ICI [41]. However, there is still insufficient evidence on whether these factors have a therapeutic effect in peritoneal metastasis. MSI-H cancers occur in gastrointestinal (colorectal, gastric, hepato-biliary) and endometrial malignancies and are caused by germline mutations in one of the DNA mismatch repair genes or somatic promoter hypermethylation of MLH. A high tumor mutation load boosts immunogenicity and ICI sensitivity [42]. After establishing a genetic signature-based predictive biomarker for systemic therapy (pembrolizumab, anti-PD-1 ICI across many tumor types), the FDA awarded its first tissue-agnostic clearance for deficient mismatch repair (dMMR)/MSI-H malignancies [43]. The FDA approval of pembrolizumab in dMMR/MSI-H was based on the KEYNOTE-158 study. The study evaluated the efficacy of pembrolizumab in patients with advanced solid tumors that had progressed on standard therapy. They found that pembrolizumab showed promising results in patients with dMMR/MSI-H solid tumors, with an overall response rate of 34.3% and a median duration of response of 24.4 months. The study suggests that pembrolizumab may be an effective treatment option for patients with dMMR/MSI-H solid tumors, including some pancreatic cancers [44].

Similarly, Kim et al. examined the therapeutic effectiveness of a STING agonist in combination with anti-PD-1 antibody therapy after implanting MC38 colon cancer cells intraperitoneally into C57BL/6 mice, causing the mice to develop PC with malignant ascites. Combination therapy with a STING agonist and anti-PD1 antibody was shown to be more effective against cancer than either drug alone. PC of colon cancer patients treated with STING agonist treatment in combination with an anti-PD1 antibody showed improved peritoneal tumor blood vascular function and enhanced anti-cancer immune response [45].

In research utilizing a newly produced highly metastatic clone of murine gastric cancer cells, YTN16P, it was shown that infusion of PD-1 mAb through the intravenous or intraperitoneal route lowered the rate of metastasis development on the mesenteric surface by 30–40% as a monotherapy [46]. Additionally, previous studies utilizing colon [40,47] or ovarian cancer cells [48,49,50] have shown that anti-PD-1 mAb may partially, but not fully, inhibit the development of PM in immunocompetent animals. Mouse models established using YTN16 and LmcMF are resistant to ICI treatment because CXCL12 derived from CAFs recruit M2 macrophages which secrete various cytokines, such as VEGF, IL-10, amphiregulin, and MMP-1 [51]. These cytokines exhaust CD8+ cells, either directly or indirectly. Furthermore, infiltration of CD8+ cells is inhibited due to the high intertumoral pressure associated with tumor fibrosis induced by CAFs. Although these models are resistant to ICI therapy, anti-CAF treatment recovered the therapeutic efficacy of the ICI [52].

Fucà et al. studied the clinical effects of peritoneal metastatic tumors and their susceptibility to ICIs in a multicenter cohort of d-MMR/MSI-H patients with 502 metastatic colorectal cancer (mCRC) in 59 metastatic gastric centers (mGC). Ascites and peritoneal metastasis reduced survival. Dual ICI (anti-CTLA-4 and PD-1) enhanced OS in mCRC patients, independent of metastasis site. Mono-ICI (anti-PD-1) therapy alone worsened survival in mCRC patients. In PM patients with ascites and d-MMR/MSI-H tumors, ICI monotherapy had no impact. The metastatic niche precludes ICI-sensitive tumors (dMMR/MSI-H) from responding [53].

Recent studies found that ICI therapy significantly improved survival in MSI/dMMR metastatic colorectal cancer and isolated PC. Eleven of forty-nine patients treated reacted fully while ten responded partially, giving a 46% iRECIST response rate. After 24.4 months, median progression-free survival (PFS) and OS were not reached. Seven of eight cytoreductive surgery patients exhibited complete pathologic response following anti-PD1 anti-CTLA-4 therapy [54]. Barraud et al. conclude that immune check-point inhibitors may help long-term PC patients without MSI/dMMR mCRC. This therapy seems to be beneficial for PC patients isolated from MSI/dMMR mCRC. Surgery for residual lesions yields the most pathological complete responses, but its efficacy is unclear [55].

NCT03311334 is a phase I/II study recruiting patients with advanced solid tumors and primary peritoneal cancer to assess the safety and effectiveness of DSP-7888 dosage emulsion in combination with an ICI (nivolumab or pembrolizumab) (RP2D). The primary aim of phase II is the assessment of the objective response rate (ORR), with other objectives including the assessment of clinical activity, safety, and tolerability [56].

As the immunological response of PC is known to be impaired, ICI monotherapy may not be effective. Therefore, an additional strategy to enhance ICI treatment response by combining it with other treatments that can activate the immunological environment of PC may be necessary.

## 4. Monoclonal Antibodies

Monoclonal antibodies (mAbs) targeting VEGF receptor such as bevacizumab and ramucirumab, which are used in the treatment of colon cancer and gastric cancer, have been shown in some studies to be helpful in the treatment of PC [57,58]. In addition, research on PC treatment using various mAbs-based ongoing clinical trials are shown in Table 2.

### 4.1. MOC31PE Immunotoxin

The monoclonal antibody called MOC31PE is derived from the *Pseudomonas* exotoxin A (PE) and targets the epithelial cell adhesion molecule (EpCAM), a transmembrane glycoprotein that is significantly overexpressed in cancerous tissue, including HGSOC, and is expressed at small levels in normal tissue [59,60]. After attaching to the EpCAM-expressing surface of cancer cells, MOC31PE kills cells by deactivating crucial cellular functions. Additionally, MOC31PE has a competitive edge over earlier anti-EpCAM antibody-based treatments due to its “simpler” mode of action, requiring just binding to EpCAM-expressing cancer cells before directly promoting cancer cell death through toxin release inside the target cells [61,62]. The therapeutic efficacy of chemotherapy-resistant cancer cells can be enhanced by MOC31PE. Recently, it was shown that patients with metastatic carcinomas who express EpCAM showed good tolerance to systemic doses of MOC31PE [63]. It was also shown that MOC31PE reduced cell survival and migration in human epithelial ovarian cancer (EOC) cell lines B76 and HOC7, indicating that MOC31PE is a promising therapeutic candidate for EOC [64].

The ImmunoPeCa experiment (NCT02219893), a phase 1 dose-escalation study carried out in 2017 [65], examined patients with peritoneal metastasis from colorectal cancer (CRC) after demonstrating anti-cancer efficacy in preclinical testing [61,64,66]. The MOC31PE immunotoxin was given intraperitoneally the day following surgery to 21 patients who had CRS/HIPEC for PC from CRC at four distinct dose levels. The medicine was found to be safe and well-tolerated, with no evidence of dose-limiting harm. Even though MOC31PE was not absorbed into the body very much, the levels in the peritoneal fluid were thought to be cytotoxic. Neutralizing antibodies were produced by all patients. The researchers came to the conclusion that these outcomes call for further study into MOC31PE immunotoxin’s effectiveness in treating PM from CRC [67], and the cytotoxic impact of MOC31PE was evaluated on newly isolated surgical EOC samples, with a 3-year overall survival (OS) estimate of 78% and a median PFS of 21 months. Ex vivo cultures of all investigated fresh EOC samples revealed that EpCAM and MOC31PE dramatically affected cell viability [68].

A study by Thorgersen et al. showed that interleukin (IL-6), interleukin-1 (IL-1), and tumor necrosis factor (TNF) levels were found to be increased following the administration of MOC31PE. This time response curve for the potent T-cell stimulator interferon (IFN) and the related chemokine interferon gamma-induced protein/chemokine (C-X-C motif) ligand 10 (IP-10) was also fascinating. These results, which are all associated with ICD (immunogenic cell death), may enhance the destruction of remaining cancer cells [69].

### 4.2. Catumaxomab

Catumaxomab is a rat-murine bispecific and trifunctional antibody that targets EpCAM and can have a long-lasting immunization effect [70,71]. In 2009, catumaxomab was approved in Europe as the first drug for malignant ascites linked to PC [72,73]. This bispecific monoclonal antibody can target immune systems and has a safe profile in clinical trials when administered intravenously (IP). Catumaxomab’s fragment-crystallizable (Fc) domain activates Fc-receptor types I, IIa, and III on NK cells, CD3+ T-cells, and EpCAM receptors, which are the substance’s two antigen-binding sites that it particularly targets. As a result of this mechanism, pro-apoptotic cytokines including IL-2, IL-12, and TNF phagocytose the targeted tumor cells, leading to cell death [74,75,76]. Numerous catumaxomab studies have demonstrated therapeutic efficacy in treating malignant ascites from primary gastric, ovarian, and CRC tumors [77,78].

In a compassionate use study conducted by Ströhlein et al., catumaxomab was administered by IP infusion to nine patients with various peritoneal surface cancers [79]. The goal of the study was to alleviate the patient’s symptoms while examining how catumaxomab treatment affected the development of long-lasting tumor immunity. A patient was given a subcutaneous injection of tumor cells that were obtained during the initial surgery four weeks following the final catumaxomab treatment. In five out of nine patients (ranging from 0.4% to 2.9%), one patient’s levels persisted for up to 110 days after the initial inoculation, indicating long-lasting immunity. Five of the nine patients with an overall average survival of 11.8 months also showed steady or partial disease regression, compared to six patients in the EVOCAPE study by Sadeghi et al. This study showed a mean overall survival of just six months [22,79]. When catumaxomab was administered as a preoperative treatment to patients with resectable gastric cancer, an acceptable toxicity profile was also demonstrated [80]. Paracentesis and catumaxomab are both effective treatments for malignant ascites that can help improve patients’ survival and quality of life [79,81].

Many ovarian cancer patients may already have peritoneal metastases at the time of their diagnosis, and the presence of a significant amount of malignant ascites accelerates the disease’s development and distention. In a trial by Burges et al., catumaxomab was used to treat ascites in 23 ovarian cancer patients who had resistant to conventional treatment. Production of ascites was significantly reduced during catumaxomab therapy in response to increasing dosages. Twenty-eight days after the last infusion, only one of twenty-three patients who received treatment needed a paracentesis, which is still nearly 2 weeks longer than is usually necessary [82,83]. In a multicenter trial conducted by Wimberger et al., 258 patients with ovarian and non-gynecologic malignancies were randomly assigned to treatment and control groups to determine the effect of catumaxomab therapy on life quality. Patient surveys were used to determine the results. Compared to paracentesis alone, treatment with catumaxomab with paracentesis considerably delayed the period until the quality of life deteriorated [84]. The therapy of malignant ascites from EpCAM+ tumors was evaluated in a randomized, multicenter study by Heiss et al. Catumaxomab significantly enhanced median puncture-free survival and time to next therapeutic intervention in the experimental group, as well as overall survival, among 258 patients with gastric cancer in this phase II/III clinical trial [75].

In a phase II study, Knödler et al. examined the use of catumaxomab (anti-EpCAM anti-CD3) in combination with systemic chemotherapy to treat PC in gastric cancer patients. The median follow-up period was 52 months. Arm A received 15 points from 5-fluorouracil, leucovorin, oxaliplatin, and docetaxel–catumaxomab (FLOT-CATU) and 16 points from FLOT alone (arm B). In total, 27 percent of PC patients in arm A and 19 percent of patients in arm B experienced complete remission (*p* = 0.69). The most common severe adverse effects of intraperitoneal CATU were vomiting (15%), fever (23%), and stomach discomfort (31%), as well as increased liver enzyme levels (gGT 31 percent bilirubin 23 percent). Comparing FLOT-CATU versus FLOT alone, adverse events tended to occur more frequently [85].

## 5. Cancer Vaccines for Peritoneal Metastasis

Therapeutic vaccines against cancer are a further immunotherapy strategy that has attracted substantial recent advancements in the intraperitoneal developments of PC. Malignant ascites have a bad prognosis and are a significant barrier to the immune system responding to vaccines. To combat this, vaccines are currently being developed and modified to specifically target ascites to enhance the quality of life for PM patients. Table 3 resume the ongoing clinical trial with cancer vaccine therapy in PC.

Cellular, viral vector, and molecular (peptide, DNA, or RNA) are the three main platforms for cancer vaccines [86]. Allogeneic tumor cell lines or autologous patient-derived tumor cells are used to create cellular vaccines [87]. Due to their functions as tumor antigen consumers, processors, and presenters, dendritic cells (DCs) are employed to create cellular cancer vaccines. Oncolytic viral vaccinations have been genetically altered to target and kill tumor cells [88]. In addition to their oncolytic effects, viral vectors also stimulate tumor-specific immune responses by providing tumor antigens through more typical T-cell priming procedures [89]. On the cell surface, major histocompatibility complex (MHC) peptides expression can be detected by T-cells [90]. For the creation of peptide-based cancer vaccines, it is important to know how peptides and T cell receptors interact with MHC. Enzymes break down short peptides, which are typically nine amino acid residues long, and immediately connect to MHC molecules, perhaps generating tolerance [91]. Longer peptides, typically 30 mer, are taken in by antigen-presenting cells (APCs), processed for MHC presentation, and result in memory CD4+ and CD8+ T cell immunological responses, which may make APCs more immunogenic [91]. DNA vaccines, often known as “naked DNA”, are closed circular DNA plasmids that encode TAAs and immunomodulatory substances intending to induce tumor-specific immune responses [92]. Despite being straightforward, secure, and quick to create, naked DNA vaccines are ineffective against target tumor cells due to low rates of transfection. mRNA vaccines, which are produced in vitro, encode an antigen or antigens, and following internalization, they express proteins that cause an immune reaction. mRNA vaccines may convey a large number of antigens and co-stimulatory signals without running the risk of infection or insertional mutagenesis, and their manufacture is rapid and affordable. However, the delivery effectiveness and stability are issues for mRNA vaccines [92].

Targeting ascites in PC has been accomplished by combining DCs with cytokine-induced killer cells (CIKs), which are cytotoxic T lymphocytes with a CD3+ CD56+ phenotype. The choice of CIKs was made based on three important criteria: they exhibit low cytotoxicity toward normal cells, no negative impact on hematopoiesis in the bone marrow, and resistance to Fas ligand-induced apoptosis. The effects of the combined treatment of DCs and CIKs include an increase in cytotoxic T cells in ascites that are driven by TNF and IFN and a reduction in immunosuppressive Tregs [93]. Similar to CAR-T cells, the method by which cancer vaccines are administered plays an important role in their dissemination. Natural killer cells (NKs) and dendritic cells (DCs) working together to fight tumors have been proven to be successful. Geller et al. demonstrated that IP- injection of IL-2-activated NK cells enhanced antitumor effects in an ovarian cancer mouse model xenograft, in contrast to systemic distribution [94]. Furthermore, Oyer et al. demonstrated that membrane-bound IL-21 and PM21 particles, produced by the plasma membrane of K562-mb21-41BBL cells, may lengthen the in vivo half-life of IP-supplied NK cells and accumulate at tumor sites in hematologic malignancies. Additionally, PM21 therapy dramatically increased human NK cell activation and proliferation in the spleen, lung, and bone marrow, supporting its potential utility as a peritoneal cavity cancer treatment [95,96]. In malignant ascites patients, Ai and coworkers assessed the efficacy and safety of IP injection of a dendritic cell vaccination in conjunction with CIKs. These patients’ quality of life significantly improved as a result of this vaccine, which was well tolerated. When this DC vaccination was administered via IP, CD3+ CD56+ CIKs expanded and displayed cytotoxic activity, whereas Treg numbers fell. Additionally, this therapy stimulated the production of IFN, which can prevent cancer cells from growing and metastasizing [93,97].

Many cycles of patient-derived type I CD4+ T helper cells (Th1) provided by IP together with the cytokines IL-2 and IFN were shown to improve the anti-tumor activity of autologous CD8+ T cells against the tumor-specific glycoform of MUC1. This was reported by Dobrzanski et al. [98,99]. In a peritoneal metastatic colon cancer murine model, Alkayyal et al. further emphasized the relevance of combining pro-inflammatory cytokine IL-12 with an oncolytic virus (Maraba MG1) for reducing tumor burden in a CT26 colon cancer model. When MG1-IL12-ICV was IP-administered to these animals, it significantly decreased tumor development, created resistance to CT26 cell reinoculation, and improved survival. Regarding the mechanism, IL-12 was effective in enticing NK cells to the tumor location for annihilation. When paired with MG1 viral proteins, these activated NK cells generated IFN, which stimulated DCs and aided in the attraction of more NK cells [100]. Similar results were discovered by Liang et al., who conducted research using a colon cancer mouse model to study the intraperitoneal administration of a recombinant plasmid that targeted FR.They discovered that the therapy significantly reduced the development of tumors and stimulated CD8+ T cells and natural killer cells to mount a response [101].

Anticancer therapy based on reovirus is currently available to patients who have developed PC resistance to existing chemotherapies since it can overcome immunosuppression by activating DCs and ultimately lead to intrinsic anti-tumor T cell activity [102]. In a work by Gujar et al., a PM murine model was produced by injection of ID8 cells into female C57BL/6 mice. This model was utilized to test reovirus-based immunotherapy (mouse ovarian carcinoma cells). The results showed prolonged survival in mice and reduced development of PC later by raising CD3+ and CD8+ T cells and inducing the Th1 cytokine IFN. It also decreased Tregs and MDSCs [103]. Internationally, clinical studies for reovirus-based cancer treatments have been conducted. For patients with primary peritoneal carcinoma and recurrent ovarian cancer, respectively, phase I and phase II trials have been finished (NCT01199263, NCT00602277) [104]. Just recently, enrolment was completed in a non-randomized phase I clinical study employing an oncolytic vaccinia virus (GL-ONC1) engineered with GFP and glucuronidase for PC patients. This vaccine, which is derived from the vaccinia virus, effectively established viral infection and reproduction when given by IP, especially in ascites liquid. The vaccine also destroyed malignant cells by releasing transgenic glucuronidase encoded by GL-ONC1 after oncolysis [105]. Furthermore, Chung and colleagues examined p53MVA in a phase I clinical study (NCT01191684) and discovered that it had a low toxic effect and was effective at enhancing p53-specific CD8 T cell responses in patients with advanced resistant colon and pancreatic cancers [106]. They also suggested that for the immune responses elicited by the p53MVA vaccination to progress to therapeutically beneficial levels, PD-1/PD-L1 suppression may be required [107].

Due to heterogeneity in cancer cells and the lack of antigenicity in recurring cancers after therapy, immunotherapy to avoid disease progression and relapse is challenging. To overcome this problem, Chianene-Bullock et al. evaluated the effectiveness and safety of a multi-peptide vaccinate which included five epitopes. This study was conducted on patients with ovarian cancer, cancer of the fallopian tubes, and peritoneal cancer. The overly high amounts of Her-2/neu, the folate binding protein (FBP), and the melanoma differentiation antigen-A1 (MAGE-A1) that are present in ovarian cancer cells led to the development of these five epitopes. The five epitopes were constrained by HLA-A1, A2, and A3 molecules. As a result, the vaccine, coupled with GM-CSF and montanide ISA-51 adjuvant, was given to those who tested positive for HLA-A1, A2, and A3. After in vitro stimulation, eight out of nine patients had a CD8 T-cell response. The combination of multipeptide vaccination with ICIs and immunological modulators should nevertheless be taken into account due to the limited T cell response [108].

## 6. CAR-T Cell Therapy for Peritoneal Carcinomatosis

### 6.1. Basic of CAR-T Cells

CAR-T cells have undergone genetic alteration to express chimeric receptors that allow them to target certain surface antigens regardless of a person’s major histocompatibility class. Although Gross et al. initially described this type of modified T cell in 1989, this technology has only evolved dramatically in the last decade, particularly for the treatment of hematologic malignancies [109]. Immunotherapy has grown in popularity since the development of CAR-T cells, which allow T cells to produce synthetic receptors against specific surface antigens and destroy tumor cells [110]. These antigens may bind to carbohydrates, glycolipids, proteoglycans, and proteins [111,112]. As a result of CAR-T cells’ therapeutic success in clinical trials of hematologic malignancies, more research is being conducted on its application to the treatment of therapy-resistant stage IV solid tumors. CAR-T cells are composed of extracellular single-chain variable fragments (scFv) of antibodies specific to the target tumor antigen and the T-cell activation domain. CAR-Ts, in contrast to specialized T-cell therapy, are MHC-independent due to the scFv component [113,114]. CAR-T cells have created a strategy for generating tumor immunity in solid tumor malignancies. Carcinoembryonic antigen (CEA) is a target for the creation of CAR-T cells to treat breast, colorectal, and gastric malignancies as solid tumors overexpress it in comparison to healthy cells [115]. In solid tumors, CAR-T targeting claudin18.2 (CLDN18.2) showed promising results including approximately 68% of patients with PC [116].

Acute lymphoblastic leukemia (ALL), B-cell lymphoma, and adult-onset ALL are among the malignancies for which CD19 is the principal target, and CAR-T cells have demonstrated encouraging treatment outcomes for hematologic malignancies. In the past five years, the FDA has approved four CD19-targeting CAR-T cell therapies: axicabtagene ciloleucel (marketed as Yescarta), tisagenlecleucel (marketed as Kymriah), lisocabtagene maraleucel (marketed as Breyanzi), and brexucabtagene autoleucel (marketed as Tecartus) [117,118,119,120].

### 6.2. Administration Route of CAR-T for Peritoneal Carcinomas

Katz et al. provided the initial description of CAR-T therapy for PC, using CEA-targeting CAR-T cells to treat colorectal PC in an animal model. The authors noticed that intraperitoneal dispersion was superior to systemic injection. Compared to systemic therapy, intraperitoneal injection resulted in a higher tumor decrease and a longer-lasting impact. These data suggest that protection against recurrence and other distant metastases may be possible [121].

Another group developed a mouse model with ovarian PC utilizing a second-generation CAR-T cell strategy that targets TAG72. The results showed intraperitoneal treatment had more benefits than intravenous administration in terms of antitumor activity and overall survival, and repeated infusions bolstered this advantage [122].

Ang et al. evaluated a mouse model of PC by employing mRNA transfection to generate CAR-T cells against EpCAM. This type of transfection had temporary effects, boosting safety in the case of adverse consequences. Due to the transient nature of the effect, repeated infusions are necessary for optimal outcomes [123]. These data suggest that local injection boosts CAR-T cell infiltration and trafficking, increases anticancer activity, increases recurrence protection, and enhances extraperitoneal antitumor efficacy while limiting systemic adverse effects. Solid tumors and PC still pose specific obstacles that must be solved. The microenvironment of the tumor, which generates an immunological and physical barrier, is the greatest impediment. The stroma of the tumor, which is rich in collagen in the extracellular matrix, is one of the components of the physical barrier. Due to the stroma, the tumor cells cannot be treated locally or systemically. However, collagenase can degrade this collagen, facilitating medication penetration [124].

In addition, intraperitoneal administration of CAR-T cells has shown antitumor activity in distant sites such as subcutaneous nodules. This is the outcome of a radiation-induced action similar to the abscopal effect, not the direct activation of CAR-T cells [125]. CAR-T cells release many tumor antigens after destroying them, causing dendritic cells to recognize them and activate a defense mechanism that is activated by antigens other than the CAR-T target [125].

Intravenous administration of third-generation CAR-T cells that target mesothelin as a therapy for gastric cancer and PC led to regression of the tumor and even elimination in a mouse model. According to reports, after two weeks, CAR-T cells remained in peripheral circulation. Therefore, CAR-T-treated animals had prolonged survival compared to untreated mice. In comparison to peritumoral injection, intravenous administration significantly accelerated tumor development in subcutaneous implants, according to the researchers [126]. Similar outcomes were seen with an intraperitoneal injection of HER2 CAR-T cells in PC due to gastric cancer, including prolonged animal survival and significantly slower tumor development compared to intraperitoneal administration of non-transduced T cells [127]. A separate group investigated ICAM-1 and compared intraperitoneal and tail vein injection techniques. A much greater tumor response was seen [128].

Early research in patients with metastatic CRC that has expanded beyond the peritoneum revealed the efficacy of this technique [129,130]. Parkhurst et al. discovered a 74–99 percent reduction in chimeric antigen receptor (CEA) levels across the board in patients with CEA (+) metastatic CRC who received CAR-T cell therapy, as well as severe transitory inflammatory colitis in every patient [130]. Katz et al. examined the effects of intraperitoneal infusion of CAR-T cells in a PC mouse model [121]. The treatment significantly reduced cancer cell growth and prolonged protection from CEA-positive peritoneal tumors, according to the study’s findings, especially when combined with antibodies that reduced the activity of regulatory T cells and myeloid-derived suppressor cells, which had multiplied in the peritoneal tumors [121].

Additionally, CD4+ Fox3+ CD25+ Tregs and granulocyte-macrophage colony-stimulating factor (GM-CSF) can increase immunosuppression in pancreatic ductal adenocarcinoma patients. Pancreatic ductal epithelial cells (PDECs) with the oncogenic KrasG12D allele generate in lymphoid organs, and GM-CSF inhibits the capacity of CAR-T cells to combat malignancies by attracting and producing Gr-1+ CD11+ (MDSCs). MDSCs release nitric oxide (NO) and deplete the environment’s arginine reserves to trigger T-cell death. In combination with immunotherapy, either inhibition of GM-CSF or MDSCs is a viable strategy for reducing tumor burden in PC patients [131,132,133].

Another surface antigen typically targeted by CAR-T therapy in ovarian, breast, and colorectal cancers is the glycosylphosphatidylinositol-anchored protein FR. In normal tissue, FR is only found on the luminal side of polarized epithelial cells. However, in tumor cells, FR levels are high, and polarization is lost. Consequently, FR that is not exposed to the bloodstream in healthy tissue is available for circulation in cancerous tissue [134]. This allows CAR-T cells delivered intravenously to target malignancies. First-generation MOV-19 CAR-T cells that target FR and CD3 intracellular signaling have failed in clinical studies because of inhomogeneous localization at tumor sites [135].

The oxygen- and nutrient-depleted malignant tumor microenvironment inhibits the proliferation and survival of CAR-T cells. In clinical investigations of CAR-T cell treatment, additional adverse effects including neurotoxicity, cytokine release syndrome, and tumor lysis syndrome resulting in hyperkalemia and hyperuricemia have been observed [115]. In addition, tumor microenvironments (TMEs) with low amounts of glucose and glutamine are significantly more detrimental to T cell activation and survival. Utilizing co-stimulatory signals such as CD28, which stimulates aerobic glycolysis, and 4-1BB, which promotes fatty acid oxidation and mitochondrial biogenesis, it is possible to increase CAR-T cell enrichment. In addition, these signals enhance effector memory T cells and extend the circulation lifespan of CAR-T cells [136,137].

Despite advancements in CAR-T cell regional distribution, immunotherapeutic applications of CAR-T cells continue to encounter difficulties as a result of immunosuppressive pathways embedded inside solid tumors. In advanced pancreatic, breast, and ovarian malignancies, elevated levels of PD-L1 drive immunosuppression. Similar to this, T cell activation in ovarian cancer ascites with malignant ascites is inhibited by the CD274 pathway, the T cell immunoglobulin and mucin domain containing 3 (TIM3)/galectin9 pathway, or the presence of PD1/PD-L or B7/H1 [138,139].

### 6.3. CAR-T Cell Studies for Peritoneal Carcinomatosis

Our knowledge of the tumor microenvironment (TME) has increased the development of CAR-T cell therapy with straight intraperitoneal administration for the treatment of PC as shown in Table 4. The transfer of T lymphocytes with the CAR (chimeric antigen receptor) gene, which is selective for tumor-associated antigens, across regional boundaries (TAAs) to the peritoneal cavity enhances the transfer of CAR-T cells to the disease location while decreasing or eliminating neurotoxicity and cytokine release syndrome. We now understand that how CAR-T cells are delivered has a profound effect on the location and regression of tumors. Katz et al. developed the injection of CAR-T cells into the regional hepatic artery for the management of hepatic malignancies caused by metastatic colorectal cancer [129]. In addition, they examined the impact of IP vs. systemic delivery of anti-CEA CAR-T cells on a C57BL6 mouse colon cancer model. Anti-CEA CAR-T cells generated from IL-2-activated murine spleen T cells were co-cultured with CEA-producing C57BL6 murine colon cancer line MC38 cells. Therapy with CAR-T cells dramatically decreased the number of MC38CEA cells relative to normal splenic T cells. In contrast to mice that have been given anti-CEA CAR-T cells by injection into the caudal vein, which resulted in a threefold reduction in tumor size, animals receiving anti-CEA CAR-T cells intraperitoneally saw a 37-fold reduction in tumor size. The therapeutic effect was amplified while anti-CEA CAR-Ts were combined with anti-PD-L1 or anti-Gr1 antibodies that inhibit MDSCs and Tregs. In addition, after 28 days as opposed to 10 days, endogenous T cells exhibited a change to an effector memory T cell phenotype (CD44+CD62L-CCR7-) in response to CAR-T treatment. Moreover, four days following intraperitoneal (IP) anti-CEA CAR-T cell infusions with daily IL-2 injections, systemic IFN levels increased significantly. These preclinical findings indicate that combination therapy may be beneficial in the procedure for treating PC [121]. Anti-CEA CAR-T cells are being looked at, and patients with breast, colorectal, and stomach cancers are currently enrolled in phase I clinical trials (NCT02349724) [115].

Song et al. showed, after completing more studies, that local application of second-generation CAR-T cells enhances long-term anti-FR CAR-T cell survival and tumor localization. The addition of the co-stimulatory signal CD137 turned these first-generation MOv-19 CAR-T cells into second-generation CAR-T cells. CD137 allows T cells containing memory and CD8+ T cells to survive. Furthermore, CD137 promotes BCl-XL expression, providing apoptosis resistance and increasing lifespan [135]. Han et al. integrated CD137 (4-1BB) into chA21 CAR-T cells to develop second-generation chA21-4-1BBz CAR-T cells that are strongly selective for cells that overexpress human epidermal growth factor receptor 2 (HER2), such as SKOV3-human ovarian cancer and NCI-N87-human gastric cancer. In a NOD-SCID mouse xenograft model, treatment with chA21-4-1BBz CAR-T cells enhanced the half-life and the concentration of CAR-T cells at the tumor site. Moreover, in this mouse model, CAR-T cell treatment of the second generation considerably reduced ascites and tumor burden [127]. Using endothelin inhibitors to halt tumor migration is an additional method for enhancing CAR-T cell localization [113,135].

CAR-T cells were examined as a potential new treatment option for ovarian cancer, which is often diagnosed in a late stage. Koneru et al. focused on the expanded extracellular domain MUC16 (MUC-16ecto) when treating advanced-stage ovarian cancer. To ensure that these CAR-Ts would activate and proliferate at the location of the tumor in the presence of immunological checkpoints, researchers engineered anti-MUC-16ecto CAR-T cells that produced IL-12. In a SCID Beige ovarian cancer xenograft model, these CAR-Ts had more efficacy than anti-MUC-16ecto CAR-T cells without an IL-12 arm to enhance antitumor activity and mouse survival when delivered intraperitoneally (IP) [140].

Hong et al. investigated the CE7 epitope of L1-CAM [141]. A separate CAR-T platform-derived T cell (CE7+R TCM) was used against the antigen in a PC model generated from human ovarian cancer (SKOV3) xenografts. To induce a substantial amount of malignant ascites, SKOV3 cells were intraperitoneally (IP) injected into NOD/scid-IL2R-null (NSG) mice. Due to its role in the establishment of treatment resistance and the progression of ovarian malignancies, L1-CAM was selected as a potential target. There was a significant reduction in tumor burden and no evidence of ascites after CE7+R TCM therapy. The T lymphocytes against L1-CAM were unable to prevent tumor recurrence due to the subsequent lowering of L1-CAM expression in recurrent illness. Significantly, the efficacy of this therapy can be enhanced by mixing CE7+R TCM cells with CAR-T cells that target distinct antigens [141,142].

According to findings by Yushu et al., the B2 CAR T cells must express a minimal quantity of fibronectin-EIIIB to be successful, which also suggests that this decreased level of fibronectin-EIIIB expression is likely connected to the poor performance of B2 CAR T cells in the MC38 model [143]. In addition, it was shown that the expression of the EIIIB+ fibronectin splice variant on neovasculature and a variety of tumor types is a target for CAR T cells that attack the stroma and vasculature of tumors and restrict tumor development. Consequently, in syngeneic immunocompetent animal models, CAR T cells based on VHH can operate as anticancer therapeutics against a range of targets. These findings demonstrate the versatility and ability of VHH-based CAR T cells to target TME and cure solid tumors [143].

To investigate its safety and effectiveness in animal models of immunocompetent mice, Diyuan et al. generated an anti-mouse EpCAM CAR (previously documented for their human counterparts, mouse EpCAM CAR-T cells display promising in vitro and in vivo antitumor activity). However, both mice with and without tumors exhibited dose-dependent toxicities following CAR-T infusion, including decreased body weight, cytokine release syndrome (CRS), and mortality [144]. A pathological examination of the patient revealed a severe and unexpected pulmonary immunopathology due to the presence of EpCAM in healthy lung tissue. In light of EpCAM CAR-T cells’ antitumor effectiveness, they conclude that EpCAM CAR-T cells used in the treatment of solid cancers may induce fatal adverse effects and should thus be assessed with caution in patients with decreased body weight, cytokine release syndrome (CRS), and mortality. Due to the presence of EpCAM in healthy lung tissue, a pathological examination of the patient revealed a severe and unexpected pulmonary immunopathology. In light of EpCAM CAR-T cells’ antitumor effectiveness, they conclude that EpCAM CAR-T cells used in the treatment of solid cancers may induce fatal adverse effects and should thus be assessed with caution in patients [144].

According to a study by A. Rodriguez-Garcia et al., folate receptor (FR) expression on tumor-associated macrophages (TAMs) leads to M2-like macrophages and is associated with the immunosuppressive profile. Intraperitoneal injection of syngeneic tumor mouse models resulted in an increase in pro-inflammatory monocytes, an inflow of endogenous CD8+ T cells that are unique to the tumor, decreased tumor development, and a rise in patient survival. Although concurrent administration of both CAR products did not increase the effectiveness of cancer-target anti-mesothelin CAR-T cells, neither did preconditioning the tumor microenvironment (TME) with FR-specific CAR-T cells [145]. These findings emphasize the pro-tumor activity of FR+ TAMs inside the tumor microenvironment (TME) and the clinical effects of TAM-depleting drugs as adjuvant treatments to standard immunotherapies that selectively target tumor antigens [145].

In a variety of syngeneic immunocompetent tumor models, PC cells derived from C57BL6 mouse colon adenocarcinoma cells were identified. Huanpeng Chen et al. reported that Sirf CAR-T (signal regulatory protein CAR-T) cells significantly enhanced survival while radically decreasing tumor burden. In addition, they observed that Sirf CAR-T cells boosted the formation of central memory T cells (TCM), increased the persistence of CAR-T cells in malignant tissue, and reduced the expression of PD-1 on the surface of CAR-T cells. Furthermore, they showed that Sirf CAR-T cells might potentially modify the tumor microenvironment by reducing myeloid-derived stem cells and boosting CD11c+ dendritic cells and M1-type macrophages in malignant cells. These data imply that the CD47 blocker SIRP-Fc boosts the anticancer effectiveness of CAR-T cells and proposes inhibiting the CD47/SIRP signaling action on CAR-T cell activity. This discovery may provide a novel strategy to treat cancer by justifying the combination of CD47 blockers and CAR-T cell therapy [146].

## 7. Comparison of Emerging Theories and Therapeutic Approaches for the PC Therapy

### 7.1. Immunotherapy Compared to Cytotoxic Chemotherapy in Peritoneal Carcinomatosis

Although conventional cytotoxic chemotherapy may partially improve the survival rate of patients with PC, its effect is limited. Currently, there is no head-to-head comparison study between immunotherapy and cytotoxic chemotherapy for PC patients. However, based on previous studies, it can be estimated that immunotherapy alone or in combination with chemotherapy may increase the survival rate of PC patients.

In the Checkmate-649 study, which focused on patients with locally advanced or metastatic gastric cancer, those with peritoneal metastasis and PD-L1 CPS ≥ 5 showed a significant improvement in overall survival with the combination of nivolumab and cytotoxic chemotherapy compared to those who received chemotherapy alone [147]. Furthermore, a study targeting patients with isolated peritoneal carcinomatosis from dMMR/MSI-H colorectal cancer showed a response rate of 46% to immune checkpoint inhibitor therapy (anti-PD1 ± anti-CTLA-4), which is a remarkable result that is difficult to achieve with conventional chemotherapy. Among the eight patients who received a combination of immune checkpoint inhibitor and chemotherapy in this study, seven showed complete remission [55]. In a study of claudin 18.2 targeting CAR-T therapy conducted in patients with advanced gastric cancer who had received extensive prior treatment, 95% of patients in the dose escalation cohort and 68% of all gastric cancer patients with PC had positive responses. This study showed promising results with a response rate of 57.1% and a 6-month survival rate of 81.2% [116]. In these studies, using ICI or CAR-T cell therapy, safety was not observed to be higher than that of conventional chemotherapy.

Based on these studies, immunotherapies have the potential to demonstrate both efficacy and safety in peritoneal carcinomatosis.

### 7.2. Emerging Theories and Therapeutic Approaches of Immunotherapy for the Treatment of Peritoneal Carcinomatosis

Recent studies have focused on the nanoparticle-based delivery of drugs into the peritoneal cavity. Nanoparticles have the potential to act as useful carriers for a wide variety of compounds and provide benefits including increased drug retention and duration and regulated drug release [148].

Several studies have explored the use of nanoparticle-based delivery systems for immunotherapy in the treatment of PC. A recent study demonstrated the potential of nanoparticle-based immunotherapy for the treatment of peritoneal metastasis in a mouse model of ovarian cancer [149]. The researchers used a nanoparticle-based delivery system (IPI549@HMP) to target and deliver anti-PD-L1 to the TME in the peritoneal cavity. The nanoparticles were designed to specifically target the tumor-associated macrophages (TAMs) in the peritoneal cavity, which play a key role in promoting tumor growth and immune suppression. The results confirmed the effective delivery of anti-PD-L1 antibodies to the TAMs in the peritoneal cavity, resulting in enhanced anti-tumor activity and improved survival in the mouse model [149]. In a recent study, researchers discovered that incorporating celastrol nanoparticles (NPs) into M1-like macrophages (NP@M1) created an effective combination therapy for cancer. These NPs helped maintain an anticancer state in the M1Φ macrophages and killed tumor cells when released. This approach could potentially treat abdominal metastasis in lung cancer, offering a promising two-pronged strategy for an otherwise incurable condition [150]. Additionally, Huang et al. discovered that apoptosis-bioinspired nanoparticles (EBN) were effectively taken up by tumor-associated macrophages (TAMs) and influenced their polarization. The EBNs also showed strong activation of the immune cascade. In fact, injecting the EBNs resulted in a greater reduction in ascites volume and a shift in immune cell subtypes compared to injections of either PBS or free TMP195 alone. Overall, this new nanodrug delivery system (NDDS) presents a promising immunotherapeutic approach for the treatment of hepatoma ascites and other malignant effusions [151].

Although progress has been made in the treatment of PC using nanoparticle-based delivery systems for immunotherapy, a deeper understanding of the effects of locoregional therapy on the human host’s physiology and immune system is necessary. It is important to investigate tumor samples from PC patients and evaluate the cytokine profiles before and after treatment to gain critical knowledge about these processes. If surgery and locoregional treatment can trigger anti-tumor immunity, these patients may benefit from additional treatment to enhance the immune response.

## 8. Conclusions

PC is a challenging and often fatal disease that has been historically resistant to conventional treatment methods such as cytoreductive surgery (CRS) and hyperthermic intraperitoneal chemotherapy (HIPEC). However, recent advances in immunotherapy offer a promising alternative for patients with PC. In particular, intra-peritoneal immunotherapy has shown great potential in breaking immunological tolerance to treat peritoneal disease. Immunotherapy treatments such as CAR-T cells, vaccine-based therapeutics, and immune checkpoint inhibitors offer new possibilities for PC treatment. Clinical trials of intraperitoneal immunotherapy are underway, and there is hope that it will become a standard of care for PC in the future. While there are still many challenges to overcome, these promising new therapies give hope for a better future for patients with peritoneal carcinomatosis. 

## Figures and Tables

**Table 1:** Ongoing clinical trials with immune checkpoint inhibitors alone therapy in PCa (source: clinicaltrials.gov (accessed on 25 February 2023).

Identifier	Trial Phase	Treatment	Cancer Condition	Administration Route	Study Status	Start-Completion Date	Autor and Country
NCT03311334	I II	Nivolumab Pembrolizumab (Anti-PD-1) DSP-7888 Dosing Emulsion	Primary Peritoneal Cancer Cancer metastases in the peritoneum	Intradermally	Completed	December 2017–November 2022	United States
NCT04442126	I, II	NM21-1480 (Anti-PDL-1/Anti-4-1BB/Anti-has Tri-Specific Antibody)	Advanced solid tumors.	Intravenously	Recruiting	August 2020–January 2025	United States
NCT03249142	I, II	Durvalumab (Anti-PD) Tremelimumab (CTLA-4) chemotherapy	Ovarian Cancer primary peritoneal or fallopian tube adenocarcinoma	Intravenously	Active, not recruiting	October 2017–April 2023	France
NCT05538091	II	Atezolizumab (Anti-PD-L1) Vismodegib	Ovarian, Fallopian Tube, and Primary Peritoneal Cancer	Intravenously	Not yet recruiting	October 2022–October 2026	United States
NCT02725489	II	Durvalumab (Anti-PD-L1) Vigil	Gynecologic cancer Breast Cancer Primary Peritoneal Carcinoma	Intravenously	Complete	Jun 2016–December 2020	United States
NCT02728830	Early Phase I	Pembrolizumab (Anti-PD-L1)	Gynecologic Cancers of Mullerian Origin	Intravenously	Active, not recruiting	Jun 2016–December 2021	United States
NCT03598270	III	Atezolizumab (Anti-PD-L1) Niraparib platinum-based doublet chemotherapy	Ovarian Cancer	Intravenously	Active, not recruiting	November 2018–January 2025	Belgium
NCT05065021	II	Dostarlimab (Anti-PD-L1) Niraparib Bevacizumab Paclitaxel	Ovarian Cancer Fallopian Tube Cancer Primary Peritoneal Cancer	Intravenously	Not yet recruiting	Jun 2022–Jun 2025	Canada
NCT04739800	II	Durvalumab (Anti-PD-L1) Cediranib olaparib	Ovarian Cancer Fallopian Tube Cancer Primary Peritoneal Cancer	Intravenously	Recruiting	April 2021–December 2023	United States
NCT02963831	I, II	Durvalumab (Anti-PD-L1) ONCOS-102 Cyclophosphamide	Colorectal Cancer Ovarian Cancer Appendiceal Cancer Biological: ONCOS-102	Intravenously	Completed	September 2017–Jun 2022	United States
NCT02659384	II	Atezolizumab (Anti-PD-L1) Bevacizumab acetylsalicylic acid	Ovarian Neoplasms Fallopian Tube or Primary Peritoneal Adenocarcinoma	Intravenously	Active, not recruiting	December 2016–February 2023	France and Netherlands
NCT02399371	II	Pembrolizumab (Anti-PD-L1)	Malignant Mesothelioma	Intravenously	Active, not recruiting	March 2015–March 2024	United States
NCT03363867	II	Atezolizumab (Anti-PD-L1) Bevacizumab Cobimetinib	Ovarian Cancer Fallopian Tube Cancer Primary Peritoneal Cancer	Intravenously	Recruiting	July 2018– February 2024	Australia
NCT04611126	I, II	Ipilimumab (anti-CTLA-4) Nivolumab (Anti-PD-1) Relatlimab Cyclophosphamide Fludarabine Phosphate	Ovarian Cancer Fallopian Tube Cancer Primary Peritoneal Cancer	Intravenously	Recruiting	April 2021–December 2023	Denmark
NCT02834013	II	Ipilimumab (anti-CTLA-4) Nivolumab (Anti-PD-1)	Peritoneal Mesothelioma Primary Peritoneal High Grade Serous Adenocarcinoma	Intravenously	Recruiting	January 2017–October 2023	United States
NCT03872947	I	Nivolumab/Pembrolizumab (Anti-PD-1) TRK-950	Solid Tumor Malignancy	Intravenously	Recruiting	April 2019–August 2024	United States
NCT03029598	I, II	Pembrolizumab (Anti-PD-1) Carboplatin	Recurrent Fallopian Tube Carcinoma Recurrent ovarian Carcinoma Recurrent Primary Peritoneal Carcinoma	Intravenously	Completed Has Results	March 2017–December 2021	United States
NCT05030246	II	Toripalimab (Anti-PD-1) Surufatinib	Refractory Metastatic Digestive System Carcinoma Primary Peritoneal Cancer	Intravenously	Recruiting	July 2021–July 2023	China
NCT04387227	II	Pembrolizumab (Anti-PD-1) Carboplatin	Recurrent Fallopian Tube Carcinoma Recurrent Ovarian Carcinoma Recurrent Primary Peritoneal Carcinoma	Intravenously	Recruiting	March 2021–April 2025	United States
NCT05648487	II	Sintilimab (anti-PD-1) Hyperthermic Intraperitoneal Chemotherapy (HIPEC)	Gastric Cancer	Intravenously	Not yet recruiting	January 2023–December 2027	China
NCT05446298	II	Pembrolizumab (anti-PD-1) ONC-392 (Anti-CTLA-4)	Ovarian Cancer High Grade Serous Adenocarcinoma of Ovary Primary Peritoneal Carcinoma Fallopian Tube Cancer	Intravenously	Recruiting	December 2022–June 2026	United States
NCT05271318	I	Pembrolizumab (anti-PD-1) TILT-123	Ovarian Carcinoma Fallopian Tube Carcinoma Primary Peritoneal Carcinoma	Intravenously	Recruiting	May 2022–March 2026	United States
NCT05581719	I, II	Nivolumab (Anti-PD-1) Allocetra-OTS	Solid Tumor Malignancy	Intravenously	Recruiting	October 2022–June 2024	Israel
NCT04042116	I, II	Nivolumab (Anti-PD-1) Lucitanib	Advanced Solid Tumor Gynecologic Cancer	Intravenously	Active, not recruiting	July 2019–January 2024	United States
NCT02571725	I, II	Tremelimumab (anti-CTLA-4) Olaparib	Ovarian Cancer Fallopian Tube Cancer Primary Peritoneal Cancer	Intravenously	Active, not recruiting	February 2016–July 2027	United States
NCT04034927	II	Tremelimumab (anti-CTLA-4) Olaparib	Ovarian Cancer Fallopian Tube Cancer Primary Peritoneal Cancer	Intravenously	Active, not recruiting	October 2019–December 2022	United States

Anti-cytotoxic T-lymphocyte antigen-4 (CTLA-4), anti-programmed cell death-1 (PD-1), anti-programmed cell death-ligand 1 (PD-L1).

**Table 2:** Active clinical studies using monoclonal antibodies for PC (retrieved on 25 February 2023) from clinicaltrials.gov.

Identifier	Trial Phase	Target	Cancer Condition	Administration Route	Start-Completion Date	Study Status	Outcomes	Country
	MOC31PE Immunotoxin (antibody MOC31)							
NCT02219893	I & II	EpCAM	Colorectal Neoplasms	Intraperitoneal	August 2014–30 May 2017	Completed	No results	Norway
	Catumaxomab							
NCT00189345	II	EpCAM, Anti-CD3	Ovarian Cancer	Intraperitoneal	May 2004–October 2005	Completed	No results	Germany
NCT00377429	II	EpCAM Anti-CD3	Ovarian Cancer	Intraperitoneal	September 2006–February 2008	Completed Has Results	- Ascites, tumor cells were eliminated - No serious adverse effects occurred.	United States
NCT01784900	II	EpCAM, Anti-CD3	Gastric Cancer	Intraperitoneal	November 2012–January 2016	Terminated	No results	France
NCT01504256	II	Anti-EpCAM Anti-CD3	Gastric Cancer	Intraperitoneal	October 2011–July 2017	completed	No results	Germany
NCT00326885	II	Anti-EpCAM, Anti-CD3	Ovarian cancer	Intraperitoneal	June 2006–August 2010	Completed Has Results	Catumaxomab extended PuFI and TTPu, im-proved ascites symptoms, and exhibited an acceptable safety profile	United States
NCT04222114	III	Anti-EpCAM, Anti-CD3	Gastric Cancer	Intraperitoneal	6 October 2020–31 August 2023	Recruiting	No results	China
NCT01246440	II	Anti-EpCAM, Anti-CD3	Ovarian cancer	Intraperitoneal	June 2010–December 2014	Completed	No results	Spain

Time to first therapeutic puncture (TTPu), puncture-free interval (PuFI), Granulocyte-macrophage colony-stimulating factor (GM-CSF).

**Table 3:** Ongoing clinical trials with cancer vaccine therapy in PC (source: clinicaltrials.gov (accessed on 25 February 2023).

Identifier	Trial Phase	Treatment	Cancer Condition	Administration Route	Study Status	Outcomes	Country
NCT02151448	I, II	Dendritic cell vaccine (αDC1 Vaccine)	Pancreas cancer	Intranodal and intradermal	Completed Has results	Well-tolerated Not acceptable for CRS/HIPEC for peritoneal metastases.	United States
NCT02275039	I	p53MVA vaccine	Ovarian cancer Fallopian Tube Primary Peritoneal Cancers	Intravenously	Completed	No results	United States
NCT00478452	I	Dendritic cell vaccine	Ovarian cancer	Intradermal	Completed	No results	United States
NCT00803569	I	ALVAC(2)-NY-ESO-1(M)/TRICOM	Ovarian cancer Fallopian Tube Primary Peritoneal Cancers	Subcutaneous	Completed Has results	NY-ESO-1 produces significant immune responses in cancer patients but has limited objective clinical responses to NY-ESO-1 expressing tumors	United States
NCT00112957	II	rV-NY-ESO-1 vaccine	Ovarian cancer Fallopian Tube Cancer Primary Peritoneal Cavity Cancer	Intradermal	Completed Has results	PFS was 21 months and OS 48 months. Vaccinated CD8+ T cells lysed NY-ESO-1-expressing tumors.	United States
NCT01673217	I	NY-ESO-1 peptide vaccine	Ovarian Fallopian Tube Cancer Primary Peritoneal Cavity Cancer	Subcutaneously	Completed	No results	United States
NCT03029403	II	DPX-Survivac	Ovarian cancer	Subcutaneously	Recruiting	No results	Canada
NCT03113487	II	P53MVA vaccine	Ovarian cancer	subcutaneously	Active, not recruiting	No results	United States
NCT03206047	I, II	DEC-205/NY-ESO-1 Fusion Protein CDX-1401	Ovarian cancer	Intravenously	Active, not recruiting	No results	United States
NCT02111941	Early Phase 1	Multi-epitope Folate Receptor Alpha-loaded Dendritic Cell Vaccine	Ovarian cancer	Intradermally	Active, not recruiting	No results	United States
NCT01606241	I	Multi-epitope Folate Receptor Alpha Peptide Vaccine	Ovarian cancer Breast Cancer	Intradermally (ID)	Completed	No results	United States
NCT02166905	I, II	DEC-205/NY-ESO-1 Fusion Protein CDX-1401	Ovarian cancer Fallopian Tube Carcinoma	Intravenously	Completed	No results	United States
NCT03332576	I	DPX-Survivac	Ovarian cancer Fallopian Tube Carcinoma	Subcutaneously	Completed	No results	Canada
NCT00616941	I	NY-ESO-1 OLP4 Montanide Poly-ICLC	Ovarian cancer Fallopian Tube Carcinoma	Subcutaneously	Completed Has results	Montanide and poly-ICLC induced NY-ESO-1-specific Th1 cells by OLP vaccination.	United States
NCT00437502	I	tumor peptide vaccine	Ovarian cancer	Intradermally subcutaneously	Completed	No results	United States
NCT01536054	I	ALVAC (2)-NY-ESO-1 (M)/TRICOM vaccine	Ovarian cancer Fallopian Tube Carcinoma Primary Peritoneal Cavity Cancer	Subcutaneously	Completed	No results	United States
NCT00857545	II	Polyvalent Antigen-KLH Conjugate Vaccine	Ovarian cancer Fallopian Tube Carcinoma Primary Peritoneal Cavity Cancer	Subcutaneously	Completed Has results	Vaccine+OPT-821 was slightly immunogenic and did not prolong PFS or OS	United States
NCT00408590	I	carcinoembryonic antigen (CEA)-expressing measles virus (MV-CEA) and oncolytic measles virus encoding thyroidal sodium iodide symporter (MV-NIS)	Ovarian cancer Primary Peritoneal Cavity Cancer	Intraperitoneally	Completed Has results	no dose-limiting toxicity, treatment-induced immunosuppression Survival rates averaged 12.15 months.	United States
NCT01416038	I	DPX-Survivac	Ovarian cancer Fallopian Tube Carcinoma Primary Peritoneal Cavity Cancer	Subcutaneously.	Completed	No results	United States
NCT00683241	I	DCVac-L	Ovarian cancer	Intradermally	Completed	No results	United States
NCT00437502	I	tumor peptide vaccine	Ovarian cancer	Intradermally Subcutaneously	Completed	No results	United States
NCT01248273	I	Globo-H-GM2-sTn-TF-Tn-KLH conjugate, plus the immunological adjuvant QS-21	Ovarian cancer Fallopian Tube Carcinoma	Subcutaneously	Completed	No results	United States
NCT02151448	I, II	DC vaccine	Appendiceal Cancer, colorectal cancer Pancreas cancer	Intradermally	Completed	No results	United States
NCT01580696	I, II	E39 peptide (100 mcg)/GM-CSF vaccine plus E39 booster	Ovarian cancer Fallopian Tube Carcinoma	Intradermally	Completed	No results	United States
NCT00006041	I	MUC1-KLH conjugate vaccine	Ovarian cancer Fallopian Tube Carcinoma	Subcutaneously	Completed	No results	United States
NCT00091273	I	ovarian cancer peptide vaccine tetanus toxoid helper peptide	Ovarian cancer	Subcutaneously Intradermally	Completed	No results	United States
NCT00066729	I	NY-ESO-1 peptide vaccine	Ovarian cancer Fallopian Tube Carcinoma	Subcutaneously	Completed Has results	Low toxicity and promotes T-cell immunity in NY-ESO-1 positive and negative tumor patients.	United States
NCT00058435	I	MOAB ACA125	Ovarian cancer Fallopian Tube Carcinoma Peritoneal Cancer	Intramuscularly, Subcutaneously	Completed	No results	United States
NCT00478387		Killed Influenza Vaccine	Ovarian Cancer Fallopian Tube, and Primary Peritoneal Cancer	Intramuscular	Completed	No results	United States
NCT00799110	II	Dendritic Cell/Tumor Fusion Vaccine	Ovarian cancer Fallopian Tube Carcinoma	Subcutaneously	Active, not recruiting	No results	United States
NCT01132014	Early Phase 1	OC-DC,	Ovarian cancer	Intranodally	Completed	No results	United States
NCT02785250	I, II	DPX-Survivac	Ovarian cancer Fallopian Tube Carcinoma	Subcutaneously	Active, not recruiting	No results	United States
NCT00398138	I	WT-1 analog peptide vaccine	Primary Peritoneal Cavity Cancer	Subcutaneously	Completed Has Results	No serious adverse effects were observed. polyvalent WT1 peptide vaccination can be safely provided to individuals with an immunological response.	United States
NCT02737787	I	WT1 Vaccine Nivolumab NY-ESO-1 Vaccine	Ovarian Cancer Fallopian Tube Primary Peritoneal Cancer	Intravenously	Active, not recruiting	No results	United States
NCT03311334	I, II	DSP-7888 Dosing Emulsion with Immune Checkpoint Inhibitors Nivolumab or Pembrolizumab	Renal Cell Carcinoma Urothelial Carcinoma Primary Peritoneal Cancer Ovarian Cancer Fallopian Tube Cancer	Intradermally	Completed	No results	United States
NCT03735589	I II	Alpha-type-1 Polarized Dendritic Cells Autologous Natural Killer Cell-like CTLs	Fallopian Tube Cancer Ovarian Cancer Primary Peritoneal Cancer	Intraperitoneal Intradermally	Not yet recruiting	No results	United States

Overall survival (OS), progression-free survival (PFS), Cytotoxic T Lymphocytes, The WT1—related Wilms tumor (WT), autologous dendritic cells pulsed with autologous oxidized tumor lysate Vaccine (OC-DC), Granulocyte-macrophage colony-stimulating factor (GM-CSF), p53-expressing modified vaccinia Ankara virus (p53MVA).

**Table 4:** Current clinical studies of CAR-T cell therapy for peritoneal carcinomatosis (source: clinicaltrials.gov) (accessed on 25 February 2023).

Identifier	Trial Phase	Target Antigen	Cancer Condition	Administration Route	Start-Completion Date	Study Status	Autor and Country
NCT03563326	I	EpCAM	Cancer Gastric with peritoneal metastasis9	Intraperitoneal	August 2018– December 2022	Recruiting	China
NCT03054298	I	huCART-meso cells	Ovarian Cancer Peritoneal Carcinoma Fallopian Tube Cancer Mesotheliomas Pleural Mesothelioma Peritoneum	Intravenous or local delivery	April 2017 –March 2025	Recruiting	United States
NCT04684459	I	HER2 and PD-L1	Peritoneal Carcinoma Metastatic	Intraperitoneal	March 2021– January 2024	Active, not recruiting	China
NCT05477927	I	VEGFR1 and PD-L1	Ovarian cancer, non-small cell lung cancer, breast cancer, gastric cancer, with peritoneal metastasis, etc.	Intrapleural or intraperitoneal	August 2022–December 2024	Active, not recruiting	China
NCT03907527	I	MUC16	Ovarian Cancer Fallopian Tube Cancer Primary peritoneal Carcinoma	Intraperitoneal and intravenously	April 2019–November 2028	Recruiting	United States
NCT03585764	I	Folate receptor-α (FRα)	Ovarian Cancer Fallopian Tube Cancer Primary peritoneal Carcinoma	Intraperitoneal	October 2018–October 2041	Recruiting	United States

Abbreviations: CAR-T, chimeric antigen receptor-expressing T cells; CEA, carcinoembryonic antigen; PD-L1, programmed cell death protein-ligand 1; MUC16, mucin 16 associated with membrane; FRα, folate receptor α; HER2, human epidermal growth factor receptor 2; L1-CAM, L1 cell adhesion molecule; NCT, national clinical trial identifier.
